# Supplementary material for: Multiscale chemogenetic dissection of fronto-temporal top-down regulation for object memory in primates
Source: Nat Commun. 2024 Jul 10;15:5369. doi: 10.1038/s41467-024-49570-w (PMC11237144; doi:10.1038/s41467-024-49570-w)
Supplement: Supplementary file 1 — Supplementary Information [file 41467_2024_49570_MOESM1_ESM.pdf]

Supplementary Information

Multiscale Chemogenetic Dissection of Fronto-temporal Top-down Regulation for Object Memory in Primates

Toshiyuki Hirabayashi, Yuji Nagai, Yuki Hori, Yukiko Hori, Kei Oyama, Koki Mimura, Naohisa Miyakawa, Haruhiko Iwaoki, Ken-ichi Inoue, Tetsuya Suhara, Masahiko Takada, Makoto Higuchi, and Takafumi Minamimoto

Supplementary Fig. 1

Supplementary Fig. 2

Supplementary Fig. 3

Supplementary Fig. 4

Supplementary Fig. 5

Supplementary Fig. 6

Supplementary Fig. 7

Supplementary References

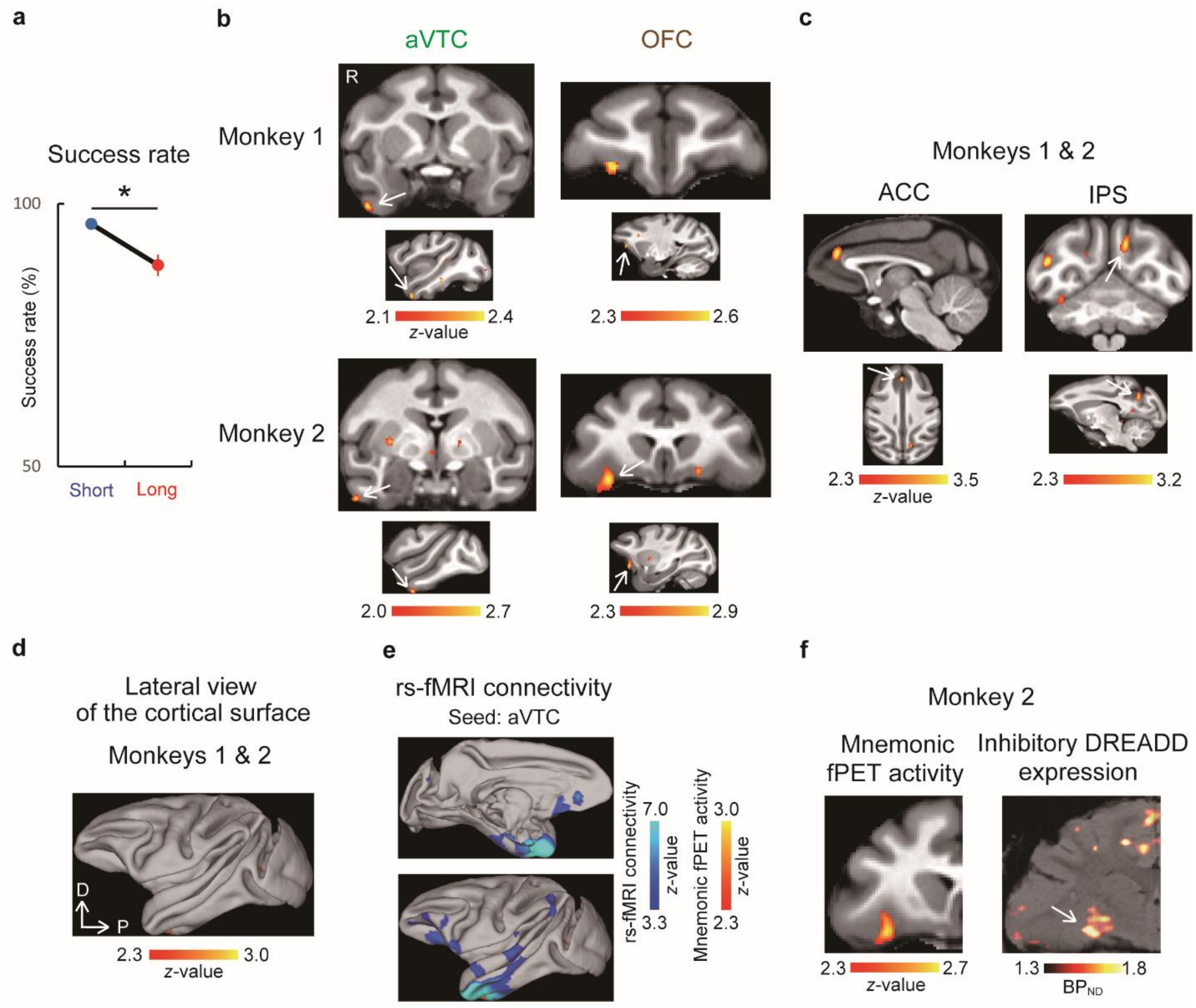

Supplementary Fig. 1 | Whole-brain fPET mapping of regions involved in retaining visual object memory.

a. DMS task performance in fPET scanning sessions for the whole-brain functional mapping of mnemonic activity. Short and Long: short- and long-delay conditions, respectively *: $P = 0.0019$, two-tailed paired t-test. Source data are provided as a Source Data file. **b.** Mnemonic fPET activity in the OFC and aVTC in each monkey. We have analyzed the spatial locations of the activations by normalizing our t-maps of fPET for each individual monkey to a publicly available macaque brain template to which a widely used standard macaque atlas[1] has been aligned[2]. According to the aforementioned atlas, the activation peaks of both monkeys shown here were located in the same anatomical area (areas 13 and TE1 for the OFC and aVTC, respectively). We also quantified the distances between the activation peaks in both monkeys and found that the distances were 6.3 and 5.5 mm for the OFC and aVTC, respectively. These measurements confirmed that, although minor spatial differences were observed, the activity was in the same anatomical area across subjects. **c.** Mnemonic fPET activity in the ACC (left) and IPS (right) in the two monkeys. **d.** Lateral view of the cortical surface showing the relative absence of mnemonic activity compared with the ventral surface (Fig. 1c, left). **e.** Medial

(top) and lateral (bottom) views of rs-fMRI connectivity with the aVTC activation site as a seed region. **f.** DREADD expression visualized with [^{11}C]DCZ-PET (right) at the fPET activation site in the OFC (left) in monkey 2.

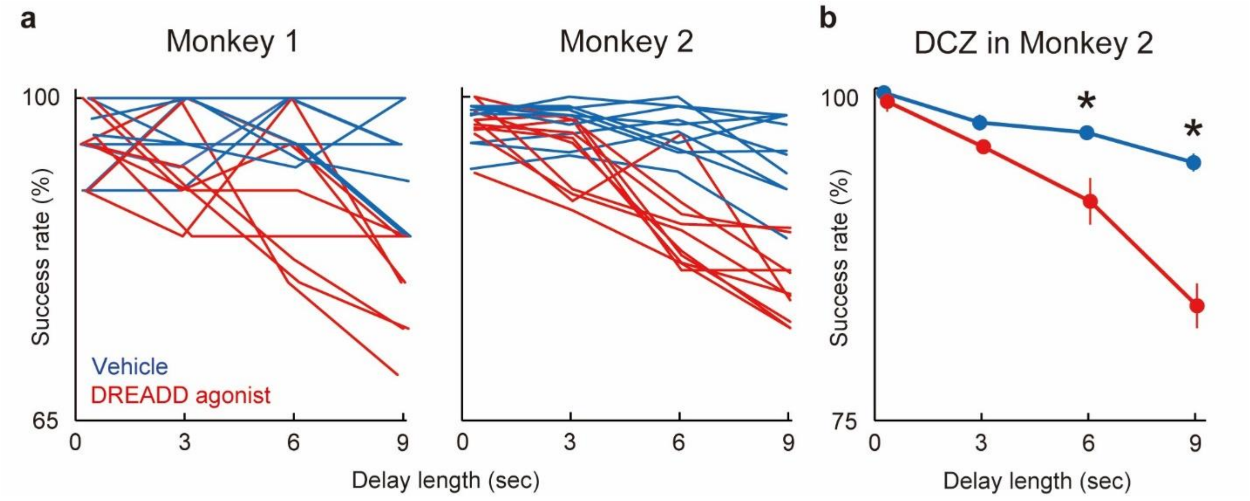

Supplementary Fig. 2 | Impact of fPET-guided OFC silencing on mnemonic behavioral performance.

a. Success rate in individual behavioral sessions for each monkey. n = 8 and 10 sessions for monkeys 1 and 2 (for each of Vehicle and DREADD agonist conditions and each delay length), respectively. **b.** Success rate of monkey 2 in vehicle (blue) and DCZ (red) conditions. *: $P = 6.5 \times 10^{-21}$ and 2.7×10^{-5} for 6 and 9 sec, respectively, two-tailed paired t-test, Bonferroni-corrected, following two-way ANOVA. n = 5 sessions for each condition (vehicle and DCZ) and each delay length. Error bars, sem. To statistically analyze the differences between the effects of CNO and DCZ within one animal (monkey 2), we performed a three-way ANOVA with the following factors: Agonist (i.e., CNO or DCZ), Silencing (i.e., vehicle or DREADD agonist), and Delay length. Although the interaction between the factors of Silencing and Delay length remained significant ($P = 3.41 \times 10^{-15}$), there was no significant interaction across all the three factors ($P = 0.18$), indicating that both agonists had similar behavioral effects as a function of the delay length, and that there were no significant differences in the effects of these agonists. Source data are provided as a Source Data file.

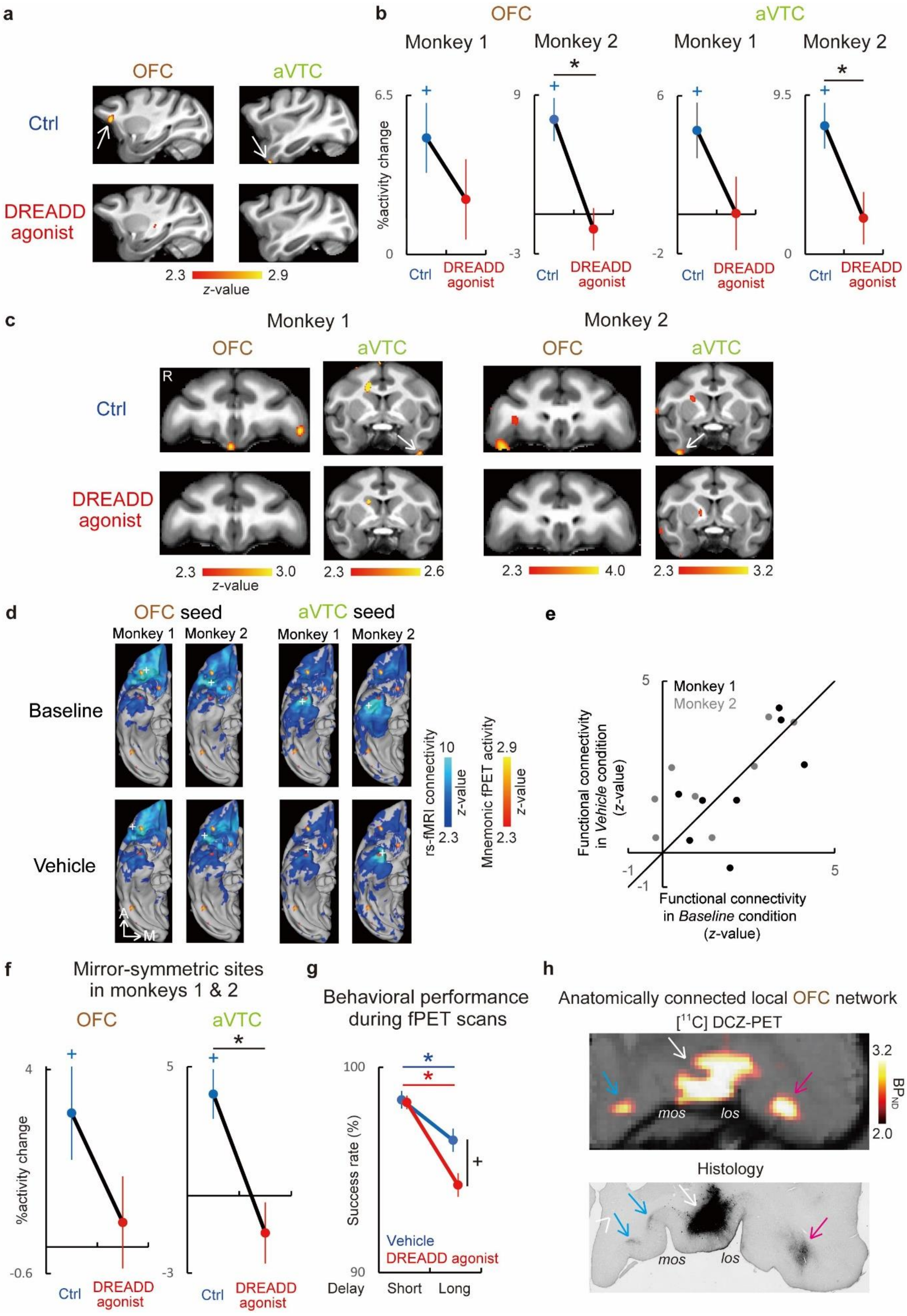

a
OFC
aVTC
Ctrl
DREADD agonist
2.3
2.9
z-value
b
OFC
aVTC
Monkey 1
Monkey 2
Monkey 1
Monkey 2
%activity change
Ctrl
DREADD agonist
c
Monkey 1
Monkey 2
OFC
aVTC
OFC
aVTC
R
Ctrl
DREADD agonist
2.3
3.0
2.3
2.6
2.3
4.0
2.3
3.2
z-value
d
OFC seed
aVTC seed
Monkey 1
Monkey 2
Monkey 1
Monkey 2
Baseline
Vehicle
rs-fMRI connectivity z-value
Mnemonic fPET activity z-value
A
M
e
Monkey 1
Monkey 2
Functional connectivity in Vehicle condition (z-value)
Functional connectivity in Baseline condition (z-value)
f
Mirror-symmetric sites in monkeys 1 & 2
OFC
aVTC
%activity change
Ctrl
DREADD agonist
g
Behavioral performance during fPET scans
Success rate (%)
Vehicle
DREADD agonist
Delay
Short
Long
h
Anatomically connected local OFC network
[11C] DCZ-PET
mos
los
BPND
Histology
mos
los

Supplementary Fig. 3 | Behavioral/imaging results of chemogenetic fPET and anatomically connected local OFC network silenced by DREADD agonist administration.

a. OFC (left) and aVTC (right) activations on the sagittal slices in the conditions of vehicle (top) and DREADD agonist (bottom) administrations in both monkeys shown in Fig. 2a. **b.** ROI analysis for the OFC (left) and aVTC (right) activation sites in Fig. 2a for each monkey. *: *P* = 0.00090 and 0.017 for OFC and aVTC, respectively, unpaired *t*-test. +: *P* = 0.0027 and 4.7×10^{-5} for OFC in monkeys 1 and 2, respectively, 0.0074 and 1.1×10^{-6} for aVTC in monkeys 1 and 2, respectively, paired *t*-test against zero. **c.** Mnemonic activity in the ventral fronto-temporal network under the conditions of vehicle (top) and DREADD agonist (bottom) administrations for each monkey. **d.** Resting-state fMRI connectivity map seeded from activation sites in the OFC and aVTC in each monkey before and after DREADD expression. For all the maps, the same fPET activation map identified in the group analysis in the vehicle condition was overlaid as the common target. White crosses, coordinates of the activation peak under each condition in each monkey. Note that both the OFC and aVTC activations in monkey 1 in the vehicle condition was observed in the opposite (left) hemisphere, and the white crosses depict left-right flipped peak coordinates for display. We analyzed the resting-state network profiles for ROIs that specifically overlap with each individual subject's mnemonic activity under both baseline (i.e., before DREADD expression) and vehicle (i.e., after DREADD expression) conditions. In these analyses, we found that when seeded from the OFC activation sites in each of the before and after DREADD expression conditions in each monkey, significant connectivity was observed at the same activation site in the aVTC that was identified in the group analysis after DREADD expression (*P* = 0.00035 and 1.2×10^{-5} for before and after DREADD expression in monkey 1, 6.7×10^{-5} and 7.3×10^{-5} for before and after DREADD expression in monkey 2). Likewise, when seeded from the aVTC activation sites in each condition in each monkey, significant connectivity was observed at the same OFC activation site that was identified in the group analysis (*P* = 0.00029 and 5.5×10^{-5} for before and after DREADD expression in monkey 1, 0.0010 and 4.0×10^{-5} for before and after DREADD expression in monkey 2). These results suggest that the activation sites before and after DREADD expression were similar to each other, at least in terms of the fronto-temporal rs-fMRI connectivity that was the particular focus of the study. **e.** Correlation between the rs-fMRI connectivity values before and after DREADD expression in each monkey. Black and gray, monkeys 1 and 2, respectively. To examine the overall consistency between the connectivity values in the two conditions, we calculated the connectivity between four OFC or aVTC activation sites (i.e., the baseline and vehicle conditions for each monkey) and the other activation sites that were identified in the group analysis after DREADD expression (i.e., the aVTC, OFC, ACC, IPS, and cEnt as shown in Fig. 3a and Supplementary Fig. 4a). As a whole, these connectivity values were significantly correlated between the conditions (*P* = 0.0021), and this significant correlation was maintained for each monkey (*P* = 0.0021 and 0.00019 for monkeys 1 and 2, respectively). These additional analyses support our claim that similar areas within the OFC and aVTC were active in both monkeys during the baseline condition (before DREADD expression) and the vehicle condition (after DREADD expression), at least in terms of rs-fMRI connectivity along the network associated with short-term visual object memory. **f.** ROI analysis for the mirror-symmetric sites in the opposite hemisphere of the activation sites

shown in Fig. 2a. *: $P = 0.00085$, unpaired t-test. +: $P = 0.019$ and 0.00027 for OFC and aVTC, respectively, paired t-test against zero **g.** Behavioral results in the chemogenetic fPET scanning sessions. *: $P = 1.3 \times 10^{-5}$ and 2.8×10^{-5} for Vehicle and DREADD agonist conditions, respectively, +: $P = 0.0197$, paired t-test, following two-way ANOVA, interaction between factors of OFC silencing and Delay length ($P = 0.0063$). **h.** Anatomical connectivity from the AAV vector injection site in the OFC consistently revealed by both [^{11}C]DCZ-PET (top) and histology (bottom) in monkey 1. Both the lateral (magenta) and medial (cyan) side of the injection site exhibited DREADD (top) and GFP (bottom) expression at the axon terminals of neurons projecting from the AAV vector injection site, thus constituting local OFC network silenced by DREADD agonist administration. *los* and *mos*, lateral and medial orbital sulci, respectively. $n = 130$ (71 and 59 for monkeys 1 and 2) and 100 (47 and 53) scans in 24 (13 and 11) and 19 (8 and 11) sessions for Ctrl and DREADD agonist conditions, respectively. Error bars are sem, statistical analyses are two-tailed, Bonferroni-corrected, and source data are provided as a Source Data file.

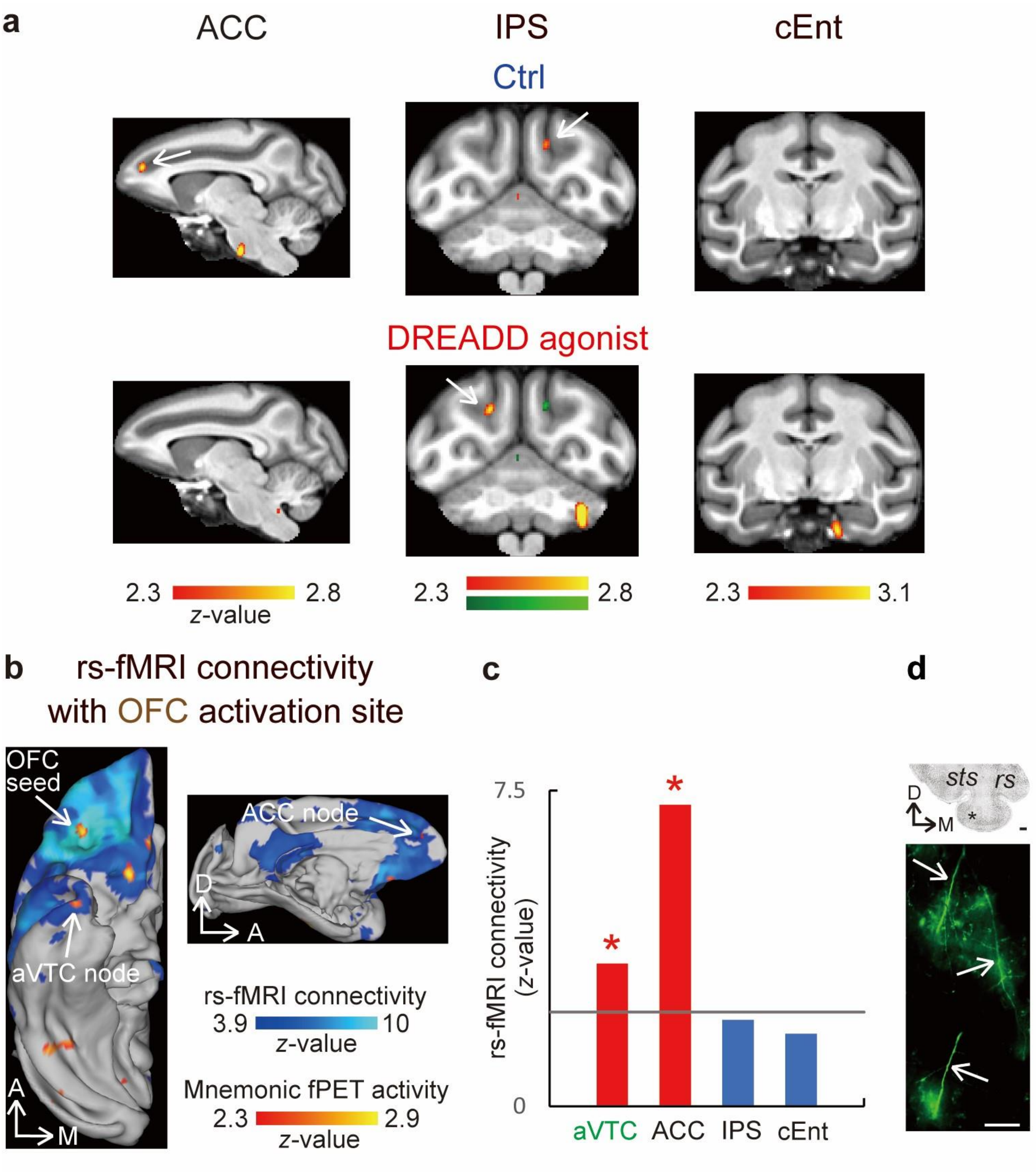

Supplementary Fig. 4 | Chemogenetic fPET results outside the ventral fronto-temporal network and rs-fMRI connectivity with the OFC node as a seed region.
a. Mnemonic activity in both monkeys outside of the ventral fronto-temporal network in the conditions of vehicle (top) and DREADD agonist (bottom) administrations. To validate the homology between the IPS activations in both hemispheres, the aforementioned macaque cortical atlas was overlaid onto our normalized activation maps to locate the IPS activations within an anatomical framework. We then compared the *y* and *z*

coordinates of the activation peaks. After adjusting for hemispheric differences by flipping the activation site in the DCZ condition from the right to the left hemisphere, the distance between the activation peaks was 2.2 mm. This panel demonstrates the proximity of the IPS activation sites in the two conditions, supporting the idea that those activation peaks were located within the same anatomical region, which was specifically identified as area PEa according to Paxinos et al., 2008[1]. Notably, previous research indicated that area PEa is associated with retrieval success signal in a recognition memory task[6]. **b.** Resting-state fMRI connectivity with the OFC activation site in Fig. 3a as a seed region. Mnemonic fPET activation map for both monkeys was overlaid. **c.** Statistical test for the strength of rs-fMRI connectivity with the OFC activation site as a seed region. Horizontal gray line, threshold for statistical significance (P = 0.05, Bonferroni-corrected). *: P =0.0014 and 3.9×10^{-13} for aVTC and ACC, respectively, one-tailed test of no correlation, Bonferroni-corrected. **d.** Histological data showing GFP-expressing axonal fibers of OFC neurons detected in the aVTC for monkey 1. Top and bottom, cortical location where GFP-expressing axonal fibers were observed shown in a Nissl-staining image and the magnified GFP image of axonal fibers, respectively. Asterisk in the top panel, approximate location of the magnified window shown in the bottom panel. Scale bar, 2 mm and 40 μm for top and bottom panels, respectively. D and M, dorsal and medial, respectively. *sts* and *rs*, superior temporal sulcus and rhinal sulcus, respectively. GFP-expressing axonal fibers of OFC neurons were detected in the aVTC, supporting our claim that DREADD-expressing OFC neurons directly connect to the aVTC, which is consistent with the findings of previous anatomical studies[3-5]. Source data are provided as a Source Data file.

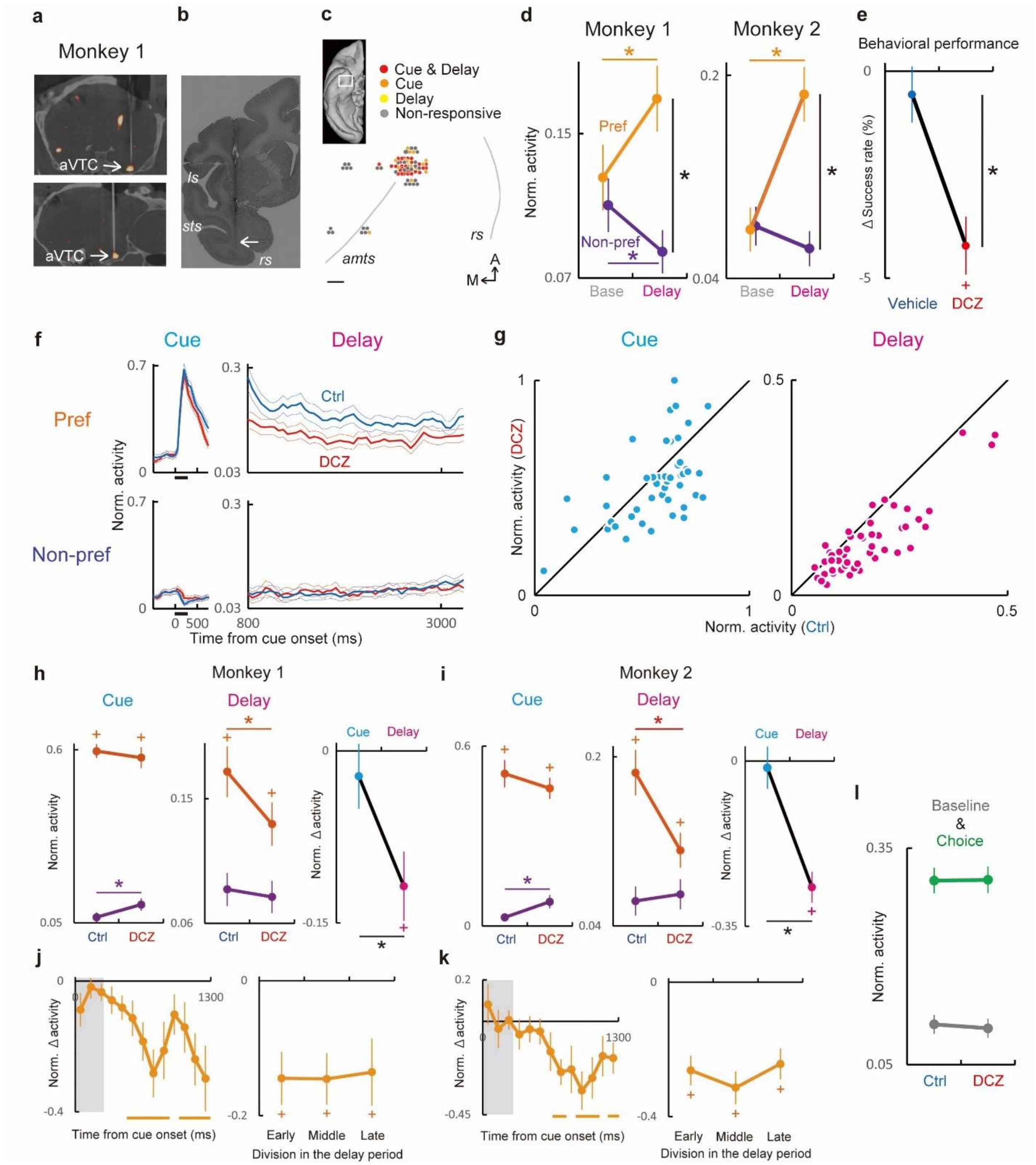

Supplementary Fig. 5 | fPET-guided chemogenetic electrophysiology in the aVTC node.

a. CT image of electrode penetration in monkey 1, on which the fPET activity map was overlaid. **b.** Histological verification of the recording tracks in monkey 2. *sts*, superior temporal sulcus; *ls*, lateral sulcus. **c.** Spatial distribution of neurons showing stimulus-selective cue and delay activity in monkey 2 around the fPET activation site in the aVTC.

Gray lines, fundus of sulci. *rs* and *amts*, rhinal sulcus and anterior middle temporal sulcus, respectively. Scale bar, 1 mm. Inset, ventral view of the left hemisphere with white rectangle depicting the approximate map location. **d.** Comparisons of activity for preferred (orange) and non-preferred (purple) stimuli between the pre-cue baseline and the delay period in each monkey. *: P = 0.00058 (Delay vs. Base for Pref), 0.0070 (Delay vs. Base for Non-pref), and 3.3×10^{-7} (Pref vs. Non-pref during Delay), paired *t*-test, Bonferroni-corrected. **e.** Percent changes in the success rate induced by vehicle (blue) and DCZ (red) administrations during neuronal recordings. *: P = 0.0018, paired *t*-test. +: $P = 1.8 \times 10^{-6}$, paired *t*-test against zero, Bonferroni-corrected. Note that because the change in success rate here is for a 3-s delay, it would be roughly equivalent to the corresponding data (i.e., 3-s delay) in Fig. 2a and Supplementary Fig. 2b, but much smaller than that for a 9 s-delay in these figure panels. **f.** Time course of neuronal activity for preferred (top) and non-preferred (bottom) stimuli directly comparing between before (blue) and during (red) OFC silencing for the same population of neurons. Thick and thin traces, mean and mean ± sem, respectively. Black horizontal bars below the traces, cue period. **g.** Comparisons of normalized firing rates of individual neurons between the control (abscissa) and OFC silencing (ordinate) conditions during the cue (left) and delay (right) periods. **h.** Left and middle, normalized neuronal activity for preferred (orange) and non-preferred (purple) stimuli in the control and OFC silencing conditions during the cue (left) and delay (right) period in monkey 1. *: P = 0.011 and 0.00027 for Cue and Delay, respectively, paired *t*-test, Bonferroni-corrected. +: $P = 2.6 \times 10^{-18}$ and 4.6×10^{-11} (Cue), 3.3×10^{-7} and 0.0083 (Delay) for Ctrl and DCZ, preferred vs. non-preferred, paired *t*-test, Bonferroni-corrected. Rightmost, OFC silencing-induced normalized change in the activity for the preferred stimulus during cue (cyan) and delay (magenta) periods in monkey 1. *: P = 0.0076, paired *t*-test. +: P = 0.0013, paired *t*-test against zero. **i.** Same as (**h**), but for monkey 2. Left and middle, *: P = 0.042 and 0.00054. +: $P = 1.4 \times 10^{-9}$ and 3.9×10^{-8} (Cue), 1.1×10^{-7} and 0.0064 (Delay). Rightmost, *: P = 0.00020, +: $P = 8.3 \times 10^{-8}$. **j.** Left, latency of the OFC silencing-induced activity reduction for the preferred stimulus for monkey 1. Orange bars, $P < 0.05$, paired *t*-test against zero. Right, OFC silencing-induced sustained activity reduction during the delay period for preferred stimulus for monkey 1. +: 0.0053 and 0.0025 for Early and Middle, respectively, paired *t*-test against zero, Bonferroni-corrected. **k.** Same as (**j**), but for monkey 2. +: 2.4×10^{-5}, 7.6×10^{-6}, and 0.00010 for Early, Middle, and Late, respectively. **l.** Comparisons of the population firing rates for the preferred stimulus between the control (left) and OFC silencing (right) conditions during the baseline (gray) and choice (green) periods. Note that although the neuronal activity during the choice period might have been affected by eye movements, the effect of OFC silencing was not observed during this period. n = 42 recording sessions for (**e**), 50 neurons showing significant stimulus selectivity during both cue and delay periods for (**f**, **g**, **i**, **l**), 29 and 21 neurons showing significant stimulus selectivity during both cue and delay periods for monkeys 1 and 2, respectively for (**d**, **h**-**k**). Error bars are sem, statistical analyses are two-tailed, and source data are provided as a Source Data file.

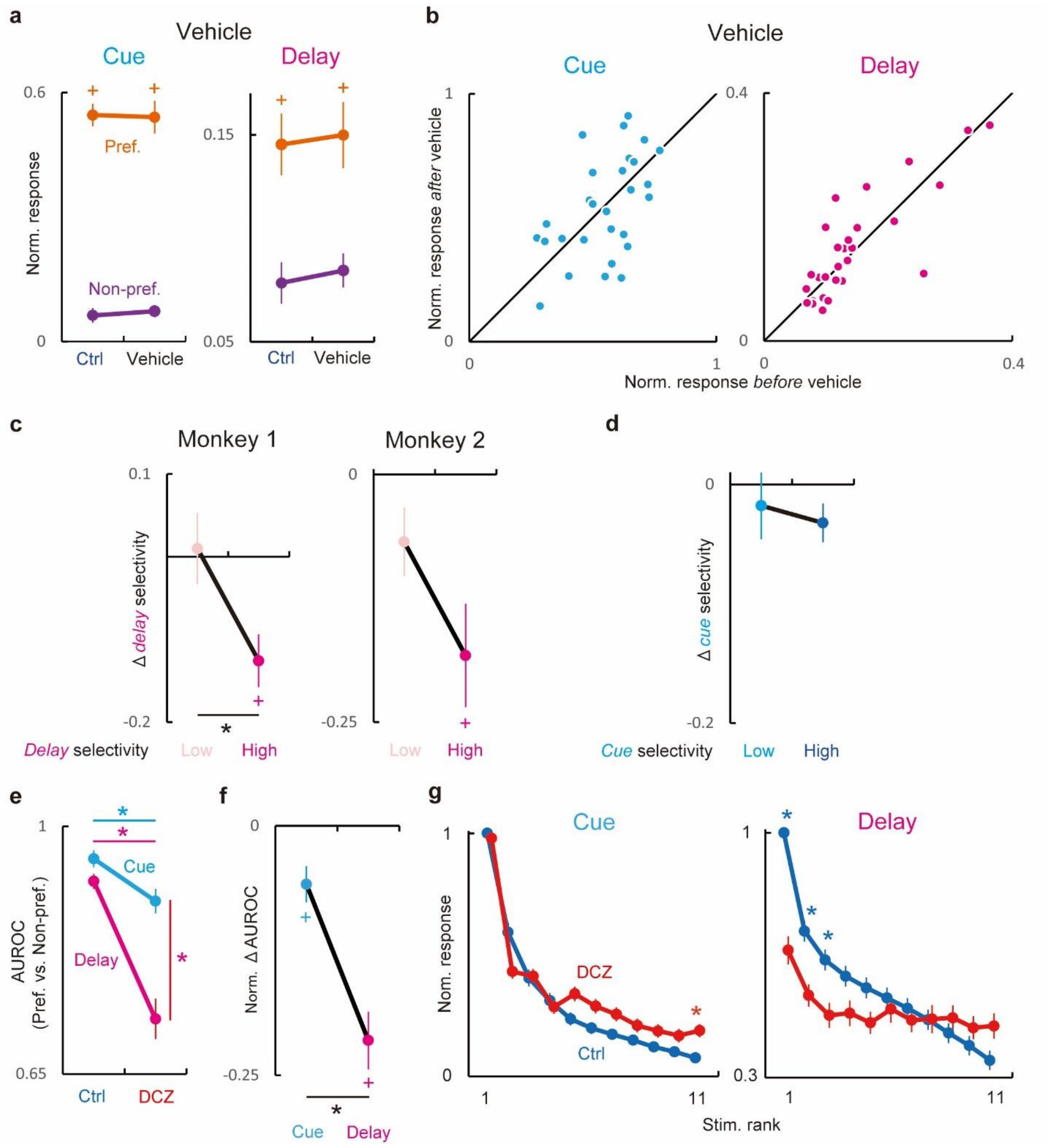

Supplementary Fig. 6 | aVTC neuronal activity in vehicle administration condition. **a.** Changes in the population firing rates induce by vehicle administration for the preferred (orange) and non-preferred (purple) stimuli during the cue (left) and delay (right) periods. +: P = 6.9 x 10^{-21} and 5.0 x 10^{-15} (Cue), 0.0012 and 0.0018 (Delay) for Ctrl and Vehicle, preferred vs. non-preferred, paired t-test following two-way ANOVA with main effect of Stimulus (P = 2.5 x 10^{-6}). **b.** Comparison of individual neuronal activities between control (abscissa) and vehicle administration (ordinate) conditions during the cue (left) and delay

(right) periods. **c.** OFC silencing-induced selectivity change of aVTC neurons with low (left) and high (right) original selectivity during the delay period in each monkey. *: P = 0.019, unpaired t-test. +: P = 0.0038 and 0.011 for monkeys 1 and 2, respectively, paired t-test against zero. **d.** OFC silencing-induced selectivity change of aVTC neurons with low (left) and high (right) original selectivity during the cue period for both monkeys. **e.** Area under the ROC curve for discriminating between the preferred and non-preferred stimuli in a trial-based manner during the cue and delay periods before and after OFC silencing. *: P = 0.0021 and 7.7×10^{-10} for Cue and Delay, respectively, Ctrl vs. DCZ, 1.4×10^{-5} for Cue vs. Delay, paired t-test, **f.** OFC silencing-induced changes in the area under the ROC curve during the cue and delay periods. *: $P = 1.4 \times 10^{-5}$, paired t-test. +: P = 0.0022 and 1.4×10^{-9} for Cue and Delay, respectively, paired t-test against zero. **g.** Normalized response to all the stimuli used in the task. For each neuron, responses to all the stimuli were normalized to that to the preferred stimulus before OFC silencing separately during the cue and delay periods. *: P = 0.024 for Cue, 1.3×10^{-9}, 6.8×10^{-5}, and 0.0025 (Stim. rank 1, 2, and 3) for Delay, paired t-test. n = 28 for (**a**-**b**), 15 (11) and 14 (10) for Low and High in monkey 1 (monkey 2) for (**c**), 25 for each of Low and High for (**d**), 50 for (**e**-**g**). Error bars are sem, statistical analyses are two-tailed, Bonferroni-corrected, and source data are provided as a Source Data file.

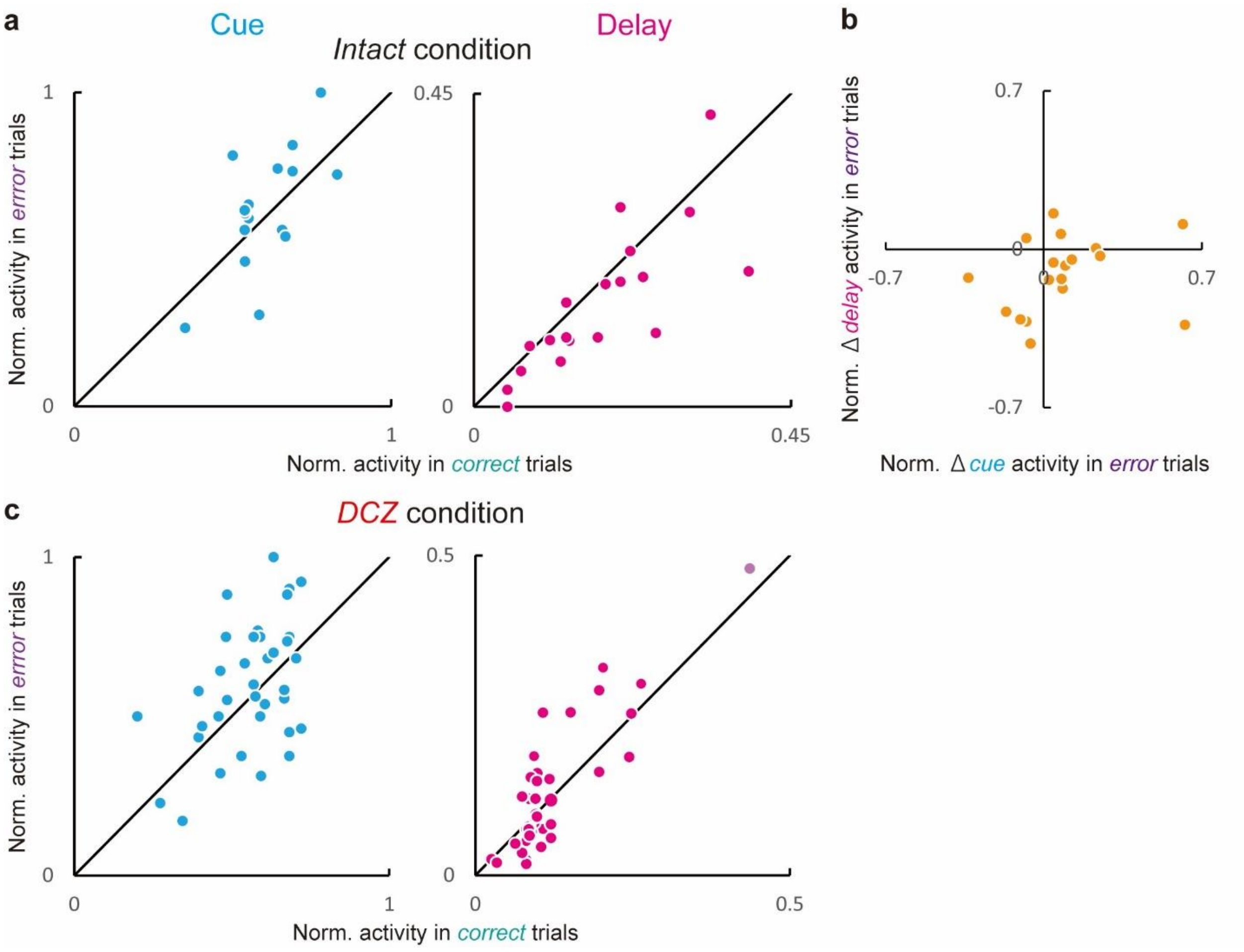

Supplementary Fig. 7 | aVTC neuronal activity in mnemonic error trials.
a. Comparison of individual aVTC neuronal activity for the preferred stimulus between correct (abscissa) and mnemonic error (ordinate) trials during the cue (left) and delay (right) periods in intact condition. These plots show that activity in the error trials decreased during the delay, but not the cue, period in each neuron. **b.** Relationships between mnemonic error-predicting decreases in the activity of each aVTC neuron for the preferred stimulus during the cue (abscissa) and delay (ordinate) periods in intact condition. If the activity decrease in the error trials during the cue period was caused by the monkeys' failure to watch the cue object correctly, and this resulted in the activity decrease during the subsequent delay period, then the changes in firing rates during these two periods would be expected to correlate with each other. However, no such correlation was identified, suggesting that the observed decrease in delay activity was unlikely to reflect such a perceptual failure. **c.** Same as (**a**), but in the condition of OFC silencing. *n* = 23 for (**a**-**b**) and 41 for (**c**). Source data are provided as a Source Data file.

Supplementary References

1. Paxinos, G., Huang, X. -F., Petrides, M., & Toga, A. W. *The Rhesus Monkey Brain in Stereotaxic Coordinates*. Academic Press, San Diego (2008).
2. Frey, S., Pandya, D.N., Chakravarty, M.M., Bailey, L., Petrides, M. & Collins, D.L. An MRI based average macaque monkey stereotaxic atlas and space (MNI monkey space). *Neuroimage* **55**, 1435-1442. (2011).
3. Saleem, K. S., Kondo, H. & Price, J. L. Complementary circuits connecting the orbital and medial prefrontal networks with the temporal, insular, and opercular cortex in the macaque monkey. *J Comp Neurol* **506**, 659-693 (2008).
4. Mohedano-Moriano, A. *et al.* Prefrontal cortex afferents to the anterior temporal lobe in the Macaca fascicularis monkey. *J Comp Neurol* **523**, 2570-2598 (2015).
5. Giarrocco, F. & Averbeck, B. B. Organization of parietoprefrontal and temporoprefrontal networks in the macaque. *J Neurophysiol* **126**, 1289-1309 (2021).
6. Miyamoto, K. *et al.* Functional differentiation of memory retrieval network in macaque posterior parietal cortex. *Neuron* **77**, 787-799 (2013).
