# Supplementary material for: Multiscale chemogenetic dissection of fronto-temporal top-down regulation for object memory in primates
Source: Nat Commun. 2024 Jul 10;15:5369. doi: 10.1038/s41467-024-49570-w (PMC11237144; doi:10.1038/s41467-024-49570-w)
Supplement: Supplementary file 3 — Reporting Summary [file 41467_2024_49570_MOESM3_ESM.pdf]

nature portfolio

Corresponding author(s): Toshiyuki Hirabayashi

Last updated by author(s): May 25, 2024

Reporting Summary

Nature Portfolio wishes to improve the reproducibility of the work that we publish. This form provides structure for consistency and transparency in reporting. For further information on Nature Portfolio policies, see our Editorial Policies and the Editorial Policy Checklist.

Statistics

For all statistical analyses, confirm that the following items are present in the figure legend, table legend, main text, or Methods section.

n/a	Confirmed	
☐	☒	The exact sample size (*n*) for each experimental group/condition, given as a discrete number and unit of measurement
☐	☒	A statement on whether measurements were taken from distinct samples or whether the same sample was measured repeatedly
☐	☒	The statistical test(s) used AND whether they are one- or two-sided<br>*Only common tests should be described solely by name; describe more complex techniques in the Methods section.*
☐	☒	A description of all covariates tested
☐	☒	A description of any assumptions or corrections, such as tests of normality and adjustment for multiple comparisons
☐	☒	A full description of the statistical parameters including central tendency (e.g. means) or other basic estimates (e.g. regression coefficient) AND variation (e.g. standard deviation) or associated estimates of uncertainty (e.g. confidence intervals)
☐	☒	For null hypothesis testing, the test statistic (e.g. *F*, *t*, *r*) with confidence intervals, effect sizes, degrees of freedom and *P* value noted<br>*Give P values as exact values whenever suitable.*
☒	☐	For Bayesian analysis, information on the choice of priors and Markov chain Monte Carlo settings
☒	☐	For hierarchical and complex designs, identification of the appropriate level for tests and full reporting of outcomes
☒	☐	Estimates of effect sizes (e.g. Cohen's *d*, Pearson's *r*), indicating how they were calculated

Our web collection on statistics for biologists contains articles on many of the points above.

Software and code

Policy information about availability of computer code

Data collection	Inquisit 3.0 (Millisecond Software), micro PET Manager 2.8 (Siemens), TDT System3 Software (Tucker Davis Technologies), Keyence BZ-X Viewer v.1.03. (Keyence), and NanoZoomer S60 (Hamamatsu Photonics K.K)
Data analysis	PMOD image analysis software 3.7 (PMOD Technologies Ltd.), Statistical Parametrical Mapping software (SPM8; Wellcome Centre for Human Neuroimaging, UCL) with MATLAB R2017 (MathWorks), FMRIB's Software Library, FMRI Expert Analysis Tool, Connectome Workbench, and Offline Sorter V4 (Plexon).

For manuscripts utilizing custom algorithms or software that are central to the research but not yet described in published literature, software must be made available to editors and reviewers. We strongly encourage code deposition in a community repository (e.g. GitHub). See the Nature Portfolio guidelines for submitting code & software for further information.

Data

Policy information about availability of data

All manuscripts must include a data availability statement. This statement should provide the following information, where applicable:

- Accession codes, unique identifiers, or web links for publicly available datasets
- A description of any restrictions on data availability
- For clinical datasets or third party data, please ensure that the statement adheres to our policy

All the rs-fMRI data used for the functional connectivity analysis is publicly available at PRIME-DE (https://fcon_1000.projects.nitrc.org/indi/indiPRIME.html). The

template macaque brain to which a widely used standard macaque atlas (Paxinos et al., 2008) has been aligned is publicly available at https://www.bic.mni.mcgill.ca/ServicesAtlases/Macaque. The rest of data reported in this paper will be shared by the corresponding author upon reasonable request.

Research involving human participants, their data, or biological material

Policy information about studies with human participants or human data. See also policy information about sex, gender (identity/presentation), and sexual orientation and race, ethnicity and racism.

Reporting on sex and gender	N/A
Reporting on race, ethnicity, or other socially relevant groupings	N/A
Population characteristics	N/A
Recruitment	N/A
Ethics oversight	N/A

Note that full information on the approval of the study protocol must also be provided in the manuscript.

Field-specific reporting

Please select the one below that is the best fit for your research. If you are not sure, read the appropriate sections before making your selection.

☒ Life sciences ☐ Behavioural & social sciences ☐ Ecological, evolutionary & environmental sciences

For a reference copy of the document with all sections, see nature.com/documents/nr-reporting-summary-flat.pdf

Life sciences study design

All studies must disclose on these points even when the disclosure is negative.

Sample size	Although no statistical methods were used to pre-determine sample sizes, our sample sizes were similar to those reported in previous related studies using macaques, which provides enough power to validate claims.
Data exclusions	No samples were excluded from the analysis.
Replication	Two macaques participated in all the experiments, and the results were consistent between the subjects, suggesting the reproducibility of our results. All attempts for replication were successful.
Randomization	The order of drug administration (DCZ/CNO or vehicle) was pseudo-random and shuffled across animals.
Blinding	The following experimental and analytical approaches acted as blinding: First, for fPET and behavioral testing data, all the sessions of data acquisition were included in analyses. Second, the locations of AAV injections for hM4Di transduction were guided by the results of fPET and rs-fMRI in a data-driven manner. And third, for electrophysiological data, all of the recorded neuronal activities with significant stimulus selectivity during both cue and delay periods before OFC silencing were included in analyses if they were continuously recorded and isolated as the same single neuronal activity in both conditions before and during OFC silencing.

Reporting for specific materials, systems and methods

We require information from authors about some types of materials, experimental systems and methods used in many studies. Here, indicate whether each material, system or method listed is relevant to your study. If you are not sure if a list item applies to your research, read the appropriate section before selecting a response.

Materials & experimental systems

n/a	Involved in the study	
☐	☒	Antibodies
☒	☐	Eukaryotic cell lines
☒	☐	Palaeontology and archaeology
☐	☒	Animals and other organisms
☒	☐	Clinical data
☒	☐	Dual use research of concern
☒	☐	Plants

Methods

n/a	Involved in the study	
☒	☐	ChIP-seq
☒	☐	Flow cytometry
☐	☒	MRI-based neuroimaging

Antibodies

Antibodies used	Rabbit anti-GFP monoclonal antibody (Thermo Fisher Scientific; GFP(G10362); Recombinant Monoclonal; 1902590,2059595, and 1965886) and biotinylated goat anti-rabbit IgG antibody (Jackson ImmunoResearch; 711-065-152; Polyclonal; 140405 and 136387)
Validation	Rabbit anti-GFP monoclonal antibody was validated by the manufacturer in the following web site: https://www.thermofisher.com/antibody/product/GFP-Tag-Antibody-Monoclonal/G10362

Animals and other research organisms

Policy information about studies involving animals; ARRIVE guidelines recommended for reporting animal research, and Sex and Gender in Research

Laboratory animals	Macaque monkeys (Macaca mulatta, male, weight: 6–8 kg, age: 5–10 years
Wild animals	N/A
Reporting on sex	Only male monkeys participated in the experiments as in most of previous studies using macaques.
Field-collected samples	N/A
Ethics oversight	All animal experiments were approved by the Animal Ethics Committee of the National Institutes for Quantum Science and Technology.

Note that full information on the approval of the study protocol must also be provided in the manuscript.

Plants

Seed stocks	N/A
Novel plant genotypes	N/A
Authentication	N/A

Magnetic resonance imaging

Experimental design

Design type	Resting-state (note that data were provided by PRIME-RE public database of macaque MRI).
Design specifications	Whole-brain BOLD fMRI data in the resting-state were collected for 53 min, 26 s from each animal. Data were collected for 20 macaques in total.
Behavioral performance measures	N/A

Acquisition

Imaging type(s)	Functional
Field strength	3 Tesla
Sequence & imaging parameters	Echo planar imaging sequence; TR, 2,000 ms; TE, 19 ms; 1,600 volumes; 36 axial slices; in-plane resolution, 2 x 2 mm; slice thickness, 2 mm; no spatial gap between slices.
Area of acquisition	Whole-brain scan was used.

Diffusion MRI ☐ Used ☒ Not used

Preprocessing

Preprocessing software	rs-fMRI data were preprocessed using FSL software. Spatial smoothing of functional images was conducted using a Gaussian kernel with the FWHM value of 2.0 mm and band-pass filtering between 0.01 and 0.1 Hz.
Normalization	The brain masks were created based on the Yerkes19 macaque brain template, which was linearly and nonlinearly registered to the individual T1-weighted structural image (T1w) using FMRIB's linear registration tool (FLIRT) and FMRIB's nonlinear registration tool (FNIRT). An average functional image was then calculated for each monkey, and was normalized to the T1w linearly and nonlinearly using FLIRT and FNIRT, respectively. Each functional image was finally normalized to the Yerkes 19 macaque template using function-to-T1w and T1w-to-template transformation matrices.
Normalization template	Yerkes 19 macaque template
Noise and artifact removal	Functional images were corrected for motion using motion correction FLIRT (MCFLIRT).
Volume censoring	N/A

Statistical modeling & inference

Model type and settings	N/A
Effect(s) tested	N/A

Specify type of analysis: ☐ Whole brain ☒ ROI-based ☐ Both

Anatomical location(s)	ROIs for ROI-to-ROI functional connectivity analyses were determined based on fPET activation sites.
Statistic type for inference (See Eklund et al. 2016)	ROI-to-ROI functional connectivity analyses were conducted, in which ROIs were determined based on fPET activation sites. The obtained z-values of functional connectivity for each seed ROI were then thresholded at P = 0.05 following correction for multiple comparisons across different target ROIs.
Correction	Bonferroni's correction across different target ROIs, for which the ROI-to-ROI functional connectivity were calculated with each seed ROI.

Models & analysis

n/a	Involved in the study
☐	☒ Functional and/or effective connectivity
☒	☐ Graph analysis
☒	☐ Multivariate modeling or predictive analysis

Functional and/or effective connectivity	Pearson correlation
